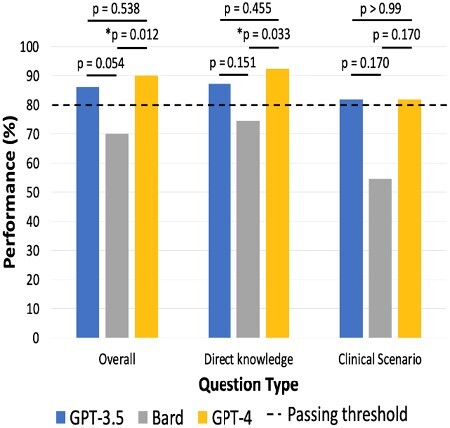# 514 GPT-4 Passes the Advanced Burn Life Support and Advanced Trauma Life Support Exams

**DOI:** 10.1093/jbcr/irae036.149

**Published:** 2024-04-17

**Authors:** Francesco Egro, Jose Antonio Arellano, Hilary Liu, Mario Alessandri-Bonetti, James Donovan, Alain C Corcos, Jenny A Ziembicki

**Affiliations:** University of Pittsburgh Medical Center, Pittsburgh, PA; University of Pittsburgh Medical Center, Pittsburgh, PA; University of Pittsburgh Medical Center, Pittsburgh, PA; University of Pittsburgh Medical Center, Pittsburgh, PA; University of Pittsburgh Medical Center, Pittsburgh, PA; University of Pittsburgh Medical Center, Pittsburgh, PA; University of Pittsburgh Medical Center, Pittsburgh, PA

## Abstract

**Introduction:**

The Advanced Burn Life Support (ABLS) and Advanced Trauma Life Support (ATLS) exams assess medical professionals’ ability to effectively evaluate, stabilize, and treat burn and trauma patients in emergency situations. Recently, there has been a growing prevalence of Artificial Intelligence (AI)-powered Natural Language Processing (NLP) chatbots, giving rise to the question of how these technologies may be integrated into medical education. To determine if LLMs could serve as clinical or educational tools for burns and trauma, this study assessed their performance on the ATLS and ABLS exams.

**Methods:**

GPT-3.5, Bard, and GPT-4 were prompted to answer the 2023 ABLS exam (50 questions) and three ATLS 10th edition exams (40 questions each). Answers produced by the AI chatbots were compared to the answer key provided by the American Burn Association (ABA) American College of Surgeons (ACS). Average exam scores were calculated, and the difference in the number of correct vs. incorrect answers between AI chatbots was evaluated using chi-square tests.

**Results:**

GPT-3.5, Bard, and GPT-4 scored 86%, 70%, and 90% on the ABLS exam, respectively. GPT-3.5 and GPT-4 scored above the passing threshold (80%), as shown in Figure 1. No difference was found between GPT-3.5 and GPT-4 (p=0.538) and between GPT-3.5 and Bard (p=0.054), despite the borderline p-value. GPT-4 performed significantly better than Bard (p=0.012). There was no difference in LLM performance on clinical scenario questions, but GPT-4 was significantly better than Bard in answering direct questions (p=0.033).

Meanwhile, on the ATLS exams, GPT-3.5 scored an average of 65%, Bard scored an average of 61.7%, and GPT-4 scored an average of 83.3%. Only GPT-4 exceeded the passing threshold (75%), as shown in Figure 2. There was no significant difference in GPT-3.5 and Bard’s average scores (65.0% vs. 61.7%, p=0.59), but GPT-4 performed significantly better than both GPT-3.5 (83.3% vs. 65.0%, p=0.0012) and Bard (83.3% vs. 61.7%, p=0.0002). There was no significant difference in GPT-3.5, Bard, or GPT-4 performance on direct vs. clinical scenario question types.

**Conclusions:**

The most recent and updated LLMs, such as GPT-4, have showcased a remarkable level of technical knowledge, to the extent of being able to successfully pass the ABLS and ATLS exams. With continued development, LLMs could become valuable resources for clinicians, enabling AI-augmented decision-making processes.

**Applicability of Research to Practice:**

This study highlights the potential applicability of advanced language models like GPT-4 as valuable resources in the burns and trauma setting, particularly in the context of enhancing clinical decision-making processes through AI-augmented tools for healthcare professionals.